# Comparison of the composition and function of the gut microbiome in herdsmen from two pasture regions, Hongyuan and Xilingol

**DOI:** 10.1002/fsn3.2290

**Published:** 2021-05-04

**Authors:** Chengcong Yang, Chuantao Peng, Hao Jin, Lijun You, Jiao Wang, Haiyan Xu, Zhihong Sun

**Affiliations:** ^1^ Key Laboratory of Dairy Biotechnology and Engineering Key Laboratory of Dairy Products Processing Inner Mongolia Agricultural University Hohhot China; ^2^ Key Laboratory of Dairy Products Processing Ministry of Agriculture and Rural Affairs Inner Mongolia Agricultural University Inner Mongolia China; ^3^ Qingdao Special Food Research Institute Qingdao Agricultural University Qingdao China

**Keywords:** *Bifidobacterium*, eating habits, gut microbiome, herdsmen, metagenomics, national minority

## Abstract

There is a close relationship between the gut microbiome and health in humans including regulation of immunity and energy metabolism. This study investigated differences in the gut microbiome of herdsmen from two regions: Hongyuan pasture in Sichuan and Xilingol pasture in Inner Mongolia. We found significant differences in the gut microbiome between the two groups. The main discriminatory species between the two groups were *Bifidobacterium longum*, *Bifidobacterium breve*, *Phascolarctobacterium succinatutens*, *Prevotella stercorea*, *Prevotella copri*, *Eubacterium biforme,* and *Fusobacterium prausnitzii*. The abundances of *Bifidobacterium longum* and *Bifidobacterium breve* were significantly lower in the gut microbiomes of Hongyuan herdsmen than in the gut microbiomes of Xilingol herdsmen. Functional metagenomic analysis showed that more genes were enriched in glycoside hydrolase and transposase in the gut microbiome of Hongyuan herdsmen compared with Xilingol herdsmen, suggesting a higher energy demand in the gut microbiome of Hongyuan herdsmen. Significantly more genes associated with glycolysis, starch degradation, and sucrose degradation were also found in the gut microbiome of Hong yuan herdsmen compared with Xilingol herdsmen. These results indicate that herdsmen from different pastoral regions had distinct gut microbiome composition and functions.

## Graphical abstract

There is a close relationship between the gut microbiome and health in humans including regulation of immunity and energy metabolism. This study investigated differences in the gut microbiome of herdsmen from two regions: Hong Yuan pasture in Sichuan and Xilingol pasture in Inner Mongolia. We found significant differences in the gut microbiome between the two groups. In addition, a higher energy demands in the gut microbiome of Hong Yuan herdsmen. Significantly more genes encoding pathways associated with glycolysis, starch degradation, and sucrose degradation were also found in the gut microbiome of Hong Yuan herdsmen compared with Xilingol herdsmen. These results support the hypothesis that the composition of the host gut microbiome is shaped by dietary intake.

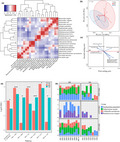

## 1 INTRODUCTION

The gut microbiome, the community of microbes living in the human gut, provides a variety of services relevant to host well‐being (Jandhyala et al., 2015). These microbes not only aid digestion and absorption of nutrients from food but also metabolize toxic substances present in the gut. Moreover, they produce essential functional amino acids and short‐chain fatty acids that are beneficial to the host immune system and metabolism (Lynch & Pedersen, 2016). Therefore, the gut microbiome is now often regarded as another “organ” of the human body (Thaiss et al., 2016). There is increasing evidence to support the hypothesis that the gut microbiome is closely related to numerous medical conditions, including colon cancer (O'keefe & hepatology, 2016), inflammatory bowel disease (Zhang & Yang, 2016), depression (Lima‐Ojeda et al., 2017), hypertension, and hyperlipidemia (Granado‐Serrano et al., 2019; Liu et al., 2017). Several factors, including dietary habits, geographical location, lifestyle, and life stage, play key roles in shaping the gut microbiome (Barone et al., 2019; Conlon & Bird, 2015; Yao et al., 2016; Yatsunenko et al., 2012).

The gut microbiome of people in urban communities is often enriched in Bacteroides, *Bifidobacterium,* and Firmicutes (Tyakht et al., 2013). One study found that 94.2% of the sequences from the gut microbiomes of children from Burkina Faso and Europe belonged to four bacterial phyla: Actinobacteria, Bacteroidetes, Firmicutes, and Proteobacteria (De Filippo et al., 2010). Pastures are grassland areas with relatively primitive ecosystems. Hongyuan and Xilingol are prominent pasture areas in China. Hongyuan pasture is located in Sichuan Province; it has a high altitude and a cold climate, and it is a pure animal husbandry county dominated by nomads. In contrast, Xilingol pasture is located in Inner Mongolia and represents a typical pastoral grassland area, where animal husbandry and herdsmen have formed independent grazing spaces. In these relatively primitive pastoral ecosystems, it is anticipated that human gut microbiomes will be rather different to those from people in urban areas and less affected by modern lifestyles and issues such as industrial pollution (De Filippo et al., 2010). Moreover, people from these pastoral communities still maintain traditional dietary habits and the diet has substantial impact on the formation and development of the gut microbiome (Li et al., 2017). However, relatively few studies have evaluated the diversity and functions of human gut microbiomes of people from these primitive pastoral ecosystems.

Second‐generation sequencing technology, represented by the Illumina sequencing platform, is a rapid, accurate, objective, and comprehensive method for evaluating microbial communities in microecosystems (Caporaso et al., 2012; Quail et al., 2012). Metagenomics uses high‐throughput sequencing to identify the collective microbial genomes present in samples without the need to isolate the microbes using traditional culture techniques (Aßhauer et al., 2015; Escobar‐Zepeda et al., 2015). In combination with metagenomic analysis, software such as The HMP Unified Metabolic Analysis Network 2 (HUMAnN2) can reveal information about the composition and potential function of microbes, and their interactions (Abubucker et al., 2017; Vernocchi et al., 2017). This technique has been widely used in the evaluation of gut microbiomes from different animals, including sheep (Al‐Masaudi et al., 2017), rats, and humans (Feng et al., 2016; Zhang et al., 2018).

In order to reveal the diversity and function of the gut microbiomes of different populations, we compared the gut microbiome of herdsmen from two local herdsman communities: Xilingol and Hongyuan, using metagenomic sequencing technology in combination with HUMAnN2. The results of this study also contribute to our understanding of the microbial diversity of healthy herdsmen living in different regions of China.

## 2 MATERIAL AND METHODS

### 2.1 Ethics statement

All experimental procedures involving human subjects were approved by the Ethics Committee of Inner Mongolia Agricultural University. A total of 18 volunteers were included in this study. All volunteers signed an informed consent form before starting the work.

### 2.2 Experimental design and sample collection

A total of 18 traditional herdsmen participated in this work, with ten subjects from the Sichuan Hongyuan Ranch (HR1 ~ HR10) and eight subjects from the Inner Mongolia Xilingol Ranch (XR1 ~ XR8) (Figure S1). None of the volunteers had any history of serious and long‐term intestinal diseases, high blood lipids, and cardiovascular diseases. Volunteers did not take antibiotic‐containing drugs in the 3 months before sampling. Sample information is shown in Table S1.

Fecal samples were collected in the morning between 1 August and 8 August 2018 and were placed in standard sampling tubes using sterile scoops before being transported to the laboratory in a cryogenic sampling box at −20℃. Upon arrival at the laboratory, the fecal samples were weighed before adding 15 ml of DNA Protected Solution (Sample Protecter for RNA/DNA) (Ambion, USA) according to the manufacturer's instructions. Each sample was then mixed evenly using a vortex mixer and stored at −20°C prior to further processing.

### 2.3 DNA extraction

The QIAamp^®^ DNA Stool Mini Kit (Qiagen, Hilden, Germany) was used for fecal DNA extraction. The quality of the extracted DNA was checked by spectrophotometry and 0.8% agarose gel electrophoresis. All eighteen DNA samples were stored in a −20°C freezer prior to further processing.

### 2.4 Shotgun metagenomic sequencing and quality control

A DNA fragment library of approximately 300 bp in length was prepared from each sample (Gao et al., 2017). DNA from samples was sequenced using an Illumina HiSeq 2000. Paired‐end readings were generated with a forward and reverse length of 101 bp each. KneadData software (Morgan & Huttenhower, 2014) was used for quality control and to remove the human sequences. The remaining high‐quality readings were used for further analysis.

### 2.5 Metagenomic bioinformatic analysis process

The extracted data were analyzed using the HUMAnN2 software tool suite (Franzosa et al., 2018). Quality‐controlled metagenomic sequence data were used for HUMAnN2 process analysis. MetaphlAn 3 (version 3.0) (Devlin et al., 2018) was used to calculate the relative abundance of groups within the microbial community. Bowtie 2 (version 2.3) software (Batut et al., 2018) and the ChocoPhlAn (version 3.0.1) (Walsh et al., 2018) database were used to check the pan‐genome nucleotide alignment at the species level. The alpha diversity was calculated using R (version 4.0.4) software (http://www.r‐project.org/) and the “Vegan” package (Oksanen, 2015). Unpaired sequences were further aligned to the UniRef 90 database using Diamond software (Ananthakrishnan et al., 2017) and translated into putative protein sequences. Finally, based on the core algorithm of HUMAnN2, genome sequences and translated protein sequences were cross compared and analyzed. In this way, the gene family, pathway coverage, and pathway abundance of the microbiomes were obtained for subsequent analysis.

### 2.6 Statistical analysis

Principal coordinates analysis (PCoA) was done using PAST (version 3.0.1) software. Differences in microbial abundance at the level of phylum, genus, species, gene, and pathways, within and between groups, were analyzed using the Wilcoxon test in R software. Dominant bacteria (those with an average relative abundance >1.0%) were analyzed using Pearson's rank correlation in R software. We used Canoco software (version 4.50) for redundancy analysis (RDA) and to screen for differentially abundant metabolic pathways and species (Gilliam & Saunders, 2003). Graphic presentations were plotted using R software (ver. 3.3.2) and Origin 2017 software (Origin Lab Corp, MA, USA).

### 2.7 Sequencing data accession numbers

The sequence data set was deposited in the National Center for Biotechnology Information (NCBI) Sequence Read Archive (SRA) database (accession number PRJNA526822, http://www.ncbi.nlm.nih.gov/bioproject/526822).

## 3 RESULTS

### 3.1 Sequence richness and diversity analysis

Whole‐genome sequencing of 18 human fecal DNA samples was performed. After quality control, the average number of double‐end reads for each sample was 14 Gb (range = 12 ~ 18; *SD* = 1.25), and they contained no human genomic DNA or linker contamination. High‐quality reads reached 252 Gb. Meanwhile, the average number of raw reads per sample was 67,886,817 (range = 56,236,152 ~ 84,706,024, *SD* = 7,208,321), and the average number of clear reads per sample was 56,815,600 (range = 49,296,736 ~ 73,394,572, *SD* = 5,502,948). After filtration and dehosting, the final number of reads remaining was 56,755,090 (range = 49,274,152 ~ 73,383,560, *SD* = 5,510,769), which were used for subsequent analysis (Table S2).

The cumulative number of species reflects the species abundance in a sample, which is an important criterion for determining whether the sample size is reasonable. As the number of samples increased, the cumulative curve for the number of species gradually stabilized (Figure S2a), indicating that the number of samples was sufficient. Although a small number of new species might have been found by increasing the number of samples taken, the bacterial diversity in the samples was still fully representative. The four α diversity indices for the gut flora of Xilingol herdsmen were higher than for Hongyuan herdsmen (Figures S2b,c). There were significant differences in, inverse Simpson, Shannon indices and Simpson between the two groups (Wilcoxon test: *p* < .05).

The phylum‐level composition of the gut microbiomes herdsmen from different pastures is shown in Figure S1. The predominant phyla (those with relative abundance >1.00%) were Firmicutes (53.54%), Bacteroidetes (32.56%), Actinobacteria (5.48%), and Proteobacteria (7.90%) for samples from Hongyuan herdsman, and 37.62%, 25.24%, 32.92%, and 3.67% for samples from Xilingol herdsman, respectively. The relative abundance of Proteobacteria in the gut microbiomes of Hongyuan herdsmen was significantly lower than in the gut microbiomes of Xilingol herdsmen (*p* = .003).

**FIGURE 1:**
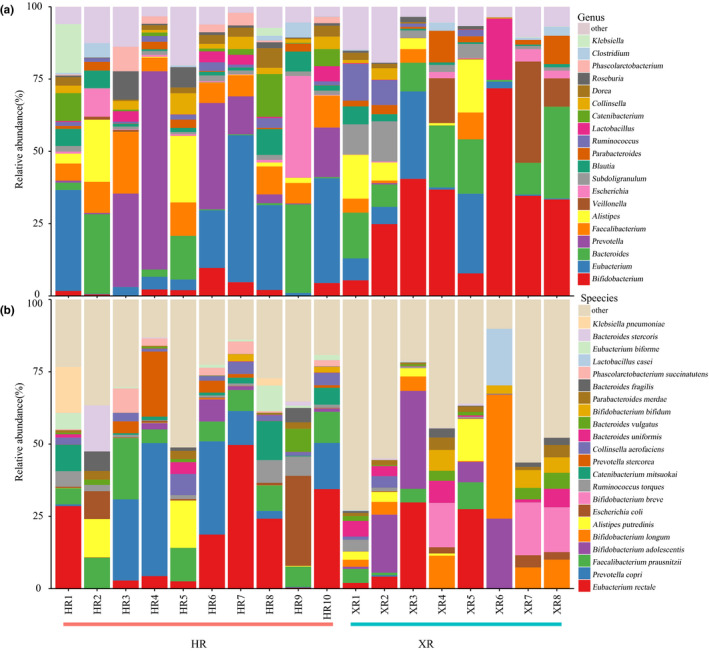
Community structure of the gut microbiome isolated from fecal samples from herdsman from two regions, at the genus (a) and species (b) level. Only dominant genera and species (more than 1% of all bacterial sequences) were shown. XR and HR represent Xilingol and Hongyuan herders, respectively

The dominant (relative abundance >1.00%) fecal bacterial genera and species in each sample is shown in Figure 1. A total of 112 genera were identified across all samples, and 20 of them had a relative abundance of >1.00%. These 20 genera represented 91.62% (range = 79.75% ~ 97.95%, *SD* = 5.41%) of the total sequences. *Bifidobacterium* and *Eubacterium* were the most prevalent genera across all samples, accounting for 30.08% of the total sequences. The genus *Eubacterium* (14.37%) was significantly more abundant in the guts of the Hongyuan herdsmen than in the guts of the Xilingol herdsmen (*p* < .001); the genus *Bifidobacterium* (15.71%) was significantly more abundant in the guts of the Xilingol herdsmen than in the guts of the Hongyuan herdsmen (*p* < .001). Other prevalent genera included *Bacteroides*, *Prevotella*, *Faecalibacterium*, *Alistipes*, *Veillonella*, *Escherichia*, *Subdoligranulum*, *Blautia*, *Parabacteroides*, *Ruminococcus,* and *Lactobacillus*, which accounted for 10.94%, 9.61%, 6.44%, 5.33%, 3.49%, 3.24%, 2.76%, 2.61%, 2.41%, 2.24%, and 2.14% of the total sequences, respectively.

A total of 226 species were identified across all samples. Among these, 21 dominant species (each with a relative abundance of >1.00%) represented 64.73% (range = 27.12% ~ 89.85%, *SD* = 16.67%) of all microbes in the gut microbiomes from both groups of herdsmen. *Eubacterium rectale* was the most common species across all samples (average of 12.74%). Although there was no overall significant difference between the two sample groups (*p* > .05), the proportion of bacteria that were *E. rectale* in the gut microbiome of Hongyuan herdsmen (16.56%) was significantly higher than that of the Xilingol herdsmen (7.96%). Other prevalent species included *Prevotella copri* (7.67%), *Faecalibacterium prausnitzii* (6.35%), *Bifidobacterium adolescentis* (5.46%), *Bifidobacterium longum* (4.67%), *Alistipes putredinis* (3.03%), *Escherichia coli* (2.98%), and *Bifidobacterium breve* (2.72%).

**FIGURE 2:**
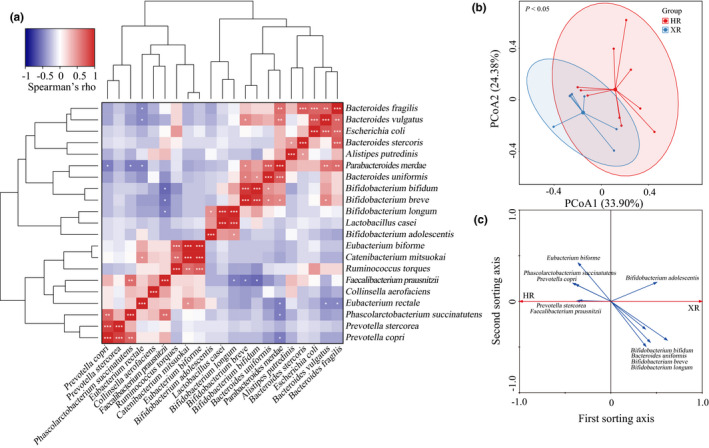
Analysis of bacterial population structure based on multivariate statistics. (a) Pearson's rank correlation among twenty‐one dominant species (more than 1.00% of all sequences). Significant correlations are represented by ***p <.001, **0.001 < *p* < .01, *0.01 < *p* < .05, respectively. Principal coordinates analysis (PCoA) score plot of bacterial microbiome structure (b) and redundancy analysis (RDA) showing key responsive species (c). XR and HR represent Xilingol and Hongyuan herders, respectively

The study further analyzed the dominant genera and species (Figure S1); there were seven significant differences between the two groups for the 20 dominant genera. With respect to species, the relative abundance of *Bifidobacterium longum* and *B. breve* was significantly lower in the gut microbiomes of Hongyuan herdsmen than in the gut microbiomes of Xilingol herdsmen (*p* < .05). Other species showed the opposite trend. Ten of the 21 dominant species were significantly more abundant in the gut microbiomes of Hongyuan herdsmen than in the gut microbiomes of Xilingol herdsmen (*p* < .05): *Catenibacterium mitsuokai*, *Phascolarctobacterium succinatutens*, *Prevotella stercorea*, *P. copri*, *Eubacterium biforme*, *F. prausnitzii*, *Collinsella aerofaciens*, and *Ruminococcus torques*. It is worth noting that the abundance of beneficial bacteria such as *Bifidobacterium* and *Lactobacillus* species was significantly higher in the gut microbiomes of Xilingol herdsmen than in the gut microbiomes of Hongyuan herdsmen (*p* < .05). Pearson's correlation analysis showed that there was a strong positive correlation among the dominant *Bifidobacterium* species, and there was a significant positive correlation between *B. longum* and *L. casei* (*p* < .001) (Figure 2a). Interestingly, *E. coli* was negatively correlated with some *Bifidobacterium* species, but positively correlated with some *Bacteroides* species.

### 3.2 Analysis of the gut microbiome based on multivariate statistical analysis

The differences in microbial community structure between the two groups (Hongyuan herdsmen and Xilingol herdsmen) were analyzed by principal coordinate analysis with Euclidean distance. PC1 and PC2 represented 33.90% and 24.38%, respectively (Figure 2b), and the gut microbiomes of the Hongyuan herdsmen and Xilingol herdsmen showed a group‐based clustering pattern (*p* < .05).

In order to determine the dominant species that caused differences in the gut microbiomes of the two groups of herdsmen, we used RDA to identify key responsive species (Figure 2c). Ten of the twenty‐one dominant species accounted for the major differences in the gut microbiomes of the two groups of herdsmen. Among the key responsive species detected, *B. adolescentis*, *P. stercorea*, and *F. prausnitzii* showed significant differences between the two groups (Figure S1).

### 3.3 Functional potential of herdsmens' gut microbiomes in different regions

**FIGURE 3:**
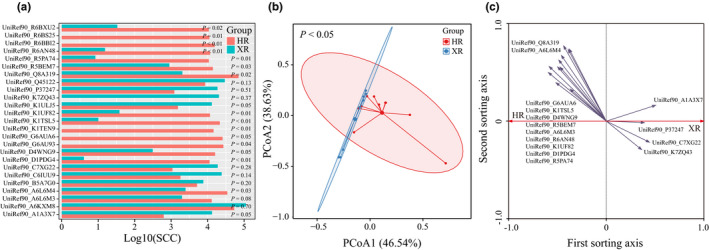
Statistical analysis at the gene level. (a) Bar chart of the 25 differentially expressed genes with the highest average abundance (*p* < .05). (b) Principal coordinates analysis (PCoA) score plot of predicted bacterial functional genes. (c) Redundancy analysis (RDA) showing key responsive genes. XR and HR represent Xilingol and Hongyuan herders, respectively. SSC represents the relative abundance of metabolic pathways. UniRef90_Q8A319: Integrase; UniRef90_A6L6M4: Mobilization protein BmgB; UniRef90_G6AUA6: Transposase, IS116/IS110/IS902 family; UniRef90_K1TSL5: Glycoside hydrolase family 24 protein (Fragment); UniRef90_K7ZQ43: Replication protein A; UniRef90_D4WNG9: Site‐specific recombinase, phage integrase family; UniRef90_C7XG22: Transposase, IS4 family; UniRef90_P37247: Transposase for insertion sequence element IS4351; UniRef90_R5BEM7: Site‐specific recombinase XerD; UniRef90_A6L6M3: Mobilization protein BmgA; UniRef90_R6AN48: 30S ribosomal protein S21; UniRef90_K1UF82: Conjugative transposon protein TraM (Fragment); UniRef90_D1PDG4: IstB‐like ATP‐binding protein; UniRef90_A1A3X7: Putative membrane protein insertion efficiency factor; UniRef90_R5PA74: 30S ribosomal protein S15

To describe the functional genomic differences between the gut microbiomes of the two groups of herdsmen, we generated a high‐quality nonredundant protein‐coding gene catalogue. A total of 1,048,575 protein‐coding genes were identified, of which 605,762 coding genes with annotated information were included in further analysis. Further, the relative abundance was calculated and sequenced, and the 25 genes with the highest abundance were selected for subsequent analysis. (Figure 3a). The results of gene annotation showed that housekeeping genes accounted for a high proportion of all genes, mainly representing protein metabolism, nucleic acid metabolism, and carbohydrate metabolism.

The UniRef90 database also noted that functional levels of the virulence‐related Integrase and Txe/YoeB family addiction module toxin were rich in both herdsmen groups. Integrase levels were significantly higher in the gut microbiomes of the Hongyuan herdsmen compared with the Xilingol herdsmen. The average relative abundance of transposase, phage integrase, mobilization protein, and ribosomal protein in the two herdsmen groups was also rich; the abundance of other subsystems, except transposase, was significantly higher in the gut microbiomes of Hongyuan herdsmen than in the gut microbiomes of Xilingol herdsmen (*p* < .05).

Similarly, PCoA and RDA analyses were done to identify distinctive features of the gut microbiomes of the two herdsmen groups, at the metagenomic level. The first and second principal coordinates accounted for 46.54% and 38.63% of the total variation, respectively (Figure 3b). Although a slight overlap was observed between the two groups, a separation trend could still be identified, resembling the results shown in Figure 2. Redundancy analysis found that 15 (out of 25) of the dominant functional genes had key responsive features that distinguished between the two herdsmen groups (Figure 3c). We further analyzed the significance of these genes involved in metabolic pathways and found significant differences in seven metabolic pathways (*p* < .05). It is also worth noting that the average relative abundances of four of these metabolic pathways were numerically higher in the gut microbiomes of Xilingol herdsmen compared with the gut microbiomes of Hongyuan herdsmen.

### 3.4 Metabolic pathways of human gut microbiomes in different regions

**FIGURE 4:**
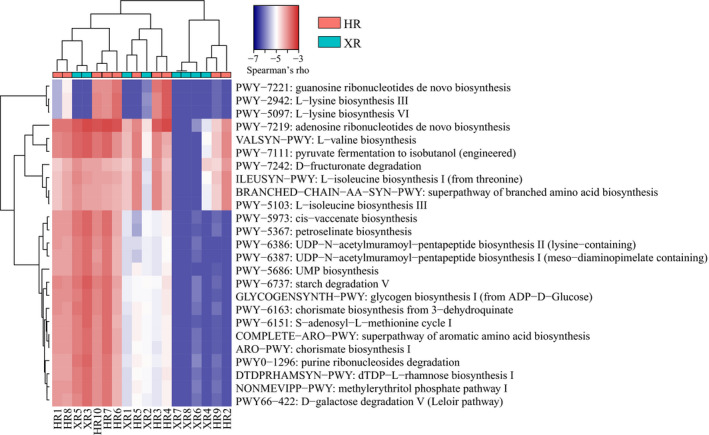
Heatmap showing the 25 most abundant MetaCyc pathways detected across the 18 metagenomic samples by HMP Unified Metabolic Analysis Network 2 (HUMAnN2) analysis. XR and HR represent Xilingol and Hongyuan herders, respectively

HUMAnN2 was used to provide a more comprehensive understanding of the potential functional role of the gut microbiomes of herdsmen. The 25 most abundant predicted metabolic pathways were associated with protein, carbohydrate, and nucleic acid metabolism (Figure 4). Using multidimensional scale analysis (MDS) on these predicted metabolic pathways, apparent differences in functional metagenomic pathway profiles were found between the two groups (Figure S3), which was supported by the results of MANOVA (*p* < .05). Significant differences between some relatively abundant metabolic pathways were noted (*p* < .05).

**FIGURE 5:**
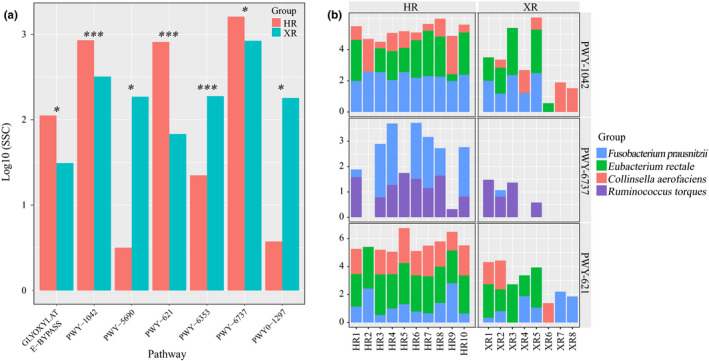
Identification of differentially abundant metabolic pathways and contributing species (a) Abundance bar graph of differentially abundant metabolic pathways in two groups. (b) Contribution of strains in different metabolic pathways. XR and HR represent Xilingol and Hongyuan herders, respectively. SSC represents the relative abundance of metabolic pathways. Significant correlations are represented by ***p < .001, **0.001 < *p* < .01, *0.01 < *p* < .05, respectively. GLYOXYLATE‐BYPASS: glyoxylate cycle, PWY‐1042: glycolysis IV (plant cytosol), PWY‐5690: TCA cycle II (plants and fungi), PWY‐621: sucrose degradation III (sucrose invertase), PWY‐6353: purine nucleotides degradation II (aerobic), PWY‐6737: starch degradation V and PWY0‐1297: superpathway of purine deoxyribonucleosides degradation

We further analyzed the 25 most abundant genes predicted in the functional metagenome and found significant differences in seven metabolic pathways between the two sample groups (*p* < .05) (Figure 5a). It is worth noting that the gut microbiome of Hongyuan herdsman had significantly more glyoxylate cycle, glycolysis IV (plant cytosol), sucrose degradation III (sucrose invertase), and starch degradation V genes, compared with the gut microbiomes of Xilingol herdsmen (*p* < .05); in contrast, the gut microbiomes of Hongyuan herdsman had significantly fewer genes related to the TCA cycle II (plants and fungi), purine nucleotides degradation II (aerobic), and the superpathway of purine deoxyribonucleosides degradation than the gut microbiomes of Xilingol herdsmen (*p* < .05). In addition, we also found an interesting phenomenon relating to glycolysis IV (plant cytosol), starch degradation V, and sucrose degradation III (sucrose invertase) involved in the metabolism of glycolysis. These pathways were more abundant in the gut microbiomes of Hongyuan herdsmen than in the gut microbiomes of Xilingol herdsmen (*p* < .05). Further studies of these three metabolic pathways revealed that they could be attributed to several species including *R*. *torques*, *E. rectale*, *F. prausnitzii*, and *C. aerofaciens*.

## 4 DISCUSSION

Increasing research on the human gut microbiome has shown that gut microbes play an important role in the digestion and absorption of nutrients from the diet (Zuo et al., 2019), as well as in regulation of immunity and metabolic functions (Li et al., 2018). The impact of differences in geographical environment on the host microbial ecosystem and health has attracted wide attention (Fallani et al., 2010). At the same time, differences in diet can impact on the composition and metabolism of the host gut microbiome greatly. The differences in the gut microbiomes of herdsmen from two different regions at the metagenomic level were analyzed and compared.

The PCoA score plot showed a clear separation trend between the gut microbiomes of herdsman from the two different regions, demonstrating that significant differences existed between the gut microbiomes of the two groups (*p* < .001). It has been shown previously that the human gut microbiome can differ between regions as a result of variation in geographical environment, diet, and altitude; with increasing altitude, microbial diversity decreases (Das et al., 2018). Hongyuan is an area of plateau pasture with a relatively high altitude (3,500 meters); this could be the main reason for the low diversity of the gut microbiome of residents of this region. Moreover, the physical distance between Hongyuan and Xilingol is >2,000 kilometers, which may also contribute to the differences in gut microbiome diversity of residents from the two locations.

Researchers often use F/B (Firmicutes/ Bacteroidetes) values to reflect the composition of the gut flora (Koliada et al., 2017). In this study, the F/B values of herdsmen from Xilingol pasture and Hongyuan were 4.10 and 7.87, respectively, and there was no significant difference between them (*p* > .05). Studies have shown that the Firmicutes/Bacteroidetes ratio of the human gut microbiome changes with age (Mariat et al., 2009). All volunteers in this study were in the same age group, which may account for the lack of a difference in the F/B values for the two groups. Meanwhile, we compared the F/B value of pastoral herders from Xilingol and Hongyuan with the F/B value of urban residents (Gupta et al., 2017) and found that the F/B value of pastoral herders was higher than the F/B value of urban residents. Some research reported that F/B values have been positively correlated with the obesity (Koliada et al., 2017); however, the Firmicutes/Bacteroidetes ratio is not a robust marker of microbiome dysbiosis associated with obesity. The reason that the F/B value of pastoral herders was higher than that of urban residents was unclear and needs future research.

The abundance of *Bifidobacterium* species in the gut is directly related to age (Arboleya et al., 2016) and is significantly higher in the guts of infants than that of adults, especially the elderly (Underwood et al., 2015). What we found is that even people of the same age group can have significant differences in the abundance of *Bifidobacterium* species in their gut flora. According to a number of reports, there is a significant relationship between the abundance *of Bifidobacterium* species in the gut and the diet (Barrett et al., 2015). The population of gut *Bifidobacterium* is significantly higher in people who regularly consume yoghurt (Suzuki et al., 2017). Herdsmen from the Xilingol area traditionally make and consume yoghurt regularly, while this is not a daily habit for herdsmen in the Hongyuan area (Sun et al., 2013). This might be the reason for the differences in abundance of *Bifidobacterium* species in the two groups. Studies have found that *Eubacterium* is potential pathogens causing cholecystitis (Berger et al., 2018), but that supplementation with probiotics can alleviate symptoms (Lau et al., 2004). Our correlation analysis showed a negative correlation between *Bifidobacterium* species and *Eubacterium* species and that the mean relative abundance of *Eubacterium* species in the gut microbiome of Xilinegol herdsman was lower than in the gut microbiome of Hongyuan herdsmen.

We found significant differences in the relative abundances of common gut bacteria, including *L. casei*, *B. adolescentis*, and *P. stercorea* between the two groups. *Lactobacillus* and *Bifidobacterium* species are some of the main bacterial species found in traditional yoghurt (Chen et al., 2018; Hill et al., 2017). The relative abundance of *P. stercorea* is known to increase following consumption of carbohydrates, because it is a good degrader of fiber and carbohydrate (Nograsek et al., 2015). The Xilingol herdsmen have a relatively simple daily diet, mainly meat and fermented dairy products (Wanni et al., 2019), while the Hongyuan herdsmen mainly consume local speciality and starchy foods (Degen et al., 2020), with a relatively low intake of dairy products. Therefore, Xilingol herdsmen consume more high‐protein foods than Hongyuan herdsmen; in support of this relationship, the abundance of *P. stercorea* was significantly lower in Xilingol herdsmen than Hongyuan herdsmen (*p <*.*05*), while *L. casei* and *B. adolescentis* (supplemented by yogurt) were significantly higher in Xilingol herdsmen than Hongyuan herdsmen.

The functional metagenomes identified in samples were predicted using HUMAnN2 software and the UniRef90 database; a large part of the metagenome was assigned to housekeeping functions, such as protein metabolism, nucleic acid metabolism, and carbohydrate metabolism. Results from PCoA and RDA showed great differences in the predicted metagenomic functions of the gut microbiomes of herdsmen from the two regions. The RDA identified key responsive genes, representing seven metabolic pathways. These genes included glycoside hydrolase and transposase, and members from the recombinase gene family. Glycoside hydrolases are enzymes that hydrolyze glycosic bonds, which exist in almost all organisms and play an important role in the hydrolysis and synthesis of sugar and glycoconjugates in organisms. The relative abundance of glycoside hydrolase genes in the gut microbiomes of Hongyuan herdsmen was significantly higher than in the gut microbiomes of Xilingol herdsmen, suggesting greater energy demands of the gut microbes from Hongyuan herdsmen. Unlike the human genome, the genomes of gut microbes contain a wide array of genes that encode glycoside hydrolases, which function to ferment indigestible polysaccharides in the colon to produce short‐chain fatty acids (SCFA) as a source of energy (Gill et al., 2006).

For Xilingol herdsmen, who traditionally eat diets based on meat and fermented dairy products, the SCFA of the gut microbiomes were estimated to account for 6%–10% of their total energy demand, while for the Hongyuan herdsmen, who's traditional diet is dominated by starches the SCFA accounts for a higher percentage of their total energy needs. This is because SCFA produced by decomposing starch are likely to contribute more energy than meat and dairy products. (Bergman, 1990). This also fully guarantees the energy supply of the Hongyuan herdsmen.

Abundance of seven of the 25 most abundant pathways was significantly different between the two groups, and most of these metabolic pathways were related to energy metabolism. The abundances of three metabolic pathways, namely glycolysis, starch degradation, and sucrose degradation, were significantly higher in the gut microbiomes of the Hongyuan herdsmen than in the Xilingol herdsmen, and they were mainly attributed to *R. torques*, *E. rectale*, *F. prausnitzii*, and *C*. *aerofaciens*. Previous studies have shown that the gut microbiome and its metabolism are affected by diet (Albenberg & Wu, 2014). The Hongyuan herdsman mainly eat local speciality, starchy foods, such as barley, while the Xilingol herdsmen eat mainly beef and mutton and yogurt and little starch and sucrose. Therefore, the abundance of their related metabolic pathways is relatively low. This also means that diet can regulate the body's energy metabolism by affecting the host's gut microbiome.

## 5 CONCLUSIONS

This study investigated differences in the gut microbiome and functions of herdsmen from two regions: Hongyuan pasture in Sichuan and Xilingol pasture in Inner Mongolia. The main discriminatory species between the two groups were *Bifidobacterium longum*, *Bifidobacterium breve*, *Phascolarctobacterium succinatutens*, *Prevotella stercorea*, *Prevotella copri*, *Eubacterium biforme,* and *Fusobacterium prausnitzii*. The abundances of *Bifidobacterium longum* and *Bifidobacterium breve* were significantly lower in the gut microbiomes of Hongy Yuan herdsmen than those of Xilingol herdsmen. Functional metagenomic analysis showed that more genes were enriched in glycoside hydrolase and transposase in the gut microbiome of Hongyuan herdsmen compared with Xilingol herdsmen, suggesting a higher energy demand in the gut microbiome of Hongy Yuan herdsmen. Significantly more genes associated with glycolysis, starch degradation, and sucrose degradation were also found in the gut microbiome of Hongyuan herdsmen compared with Xilingol herdsmen. These results indicate that herdsmen from different pastoral regions had distinct gut microbiome composition and functions.

## CONFLICT OF INTEREST

The authors declare that they have no conflicts of interest.

## Supporting information

Table S1

Table S2

Figure S1

Figure S2

Figure S3
