# Supplementary figures and images for: Comparison of the composition and function of the gut microbiome in herdsmen from two pasture regions, Hongyuan and Xilingol
Source: Food Sci Nutr. 2021 May 4;9(6):3258–68. doi: 10.1002/fsn3.2290 (PMC8194741; doi:10.1002/fsn3.2290)

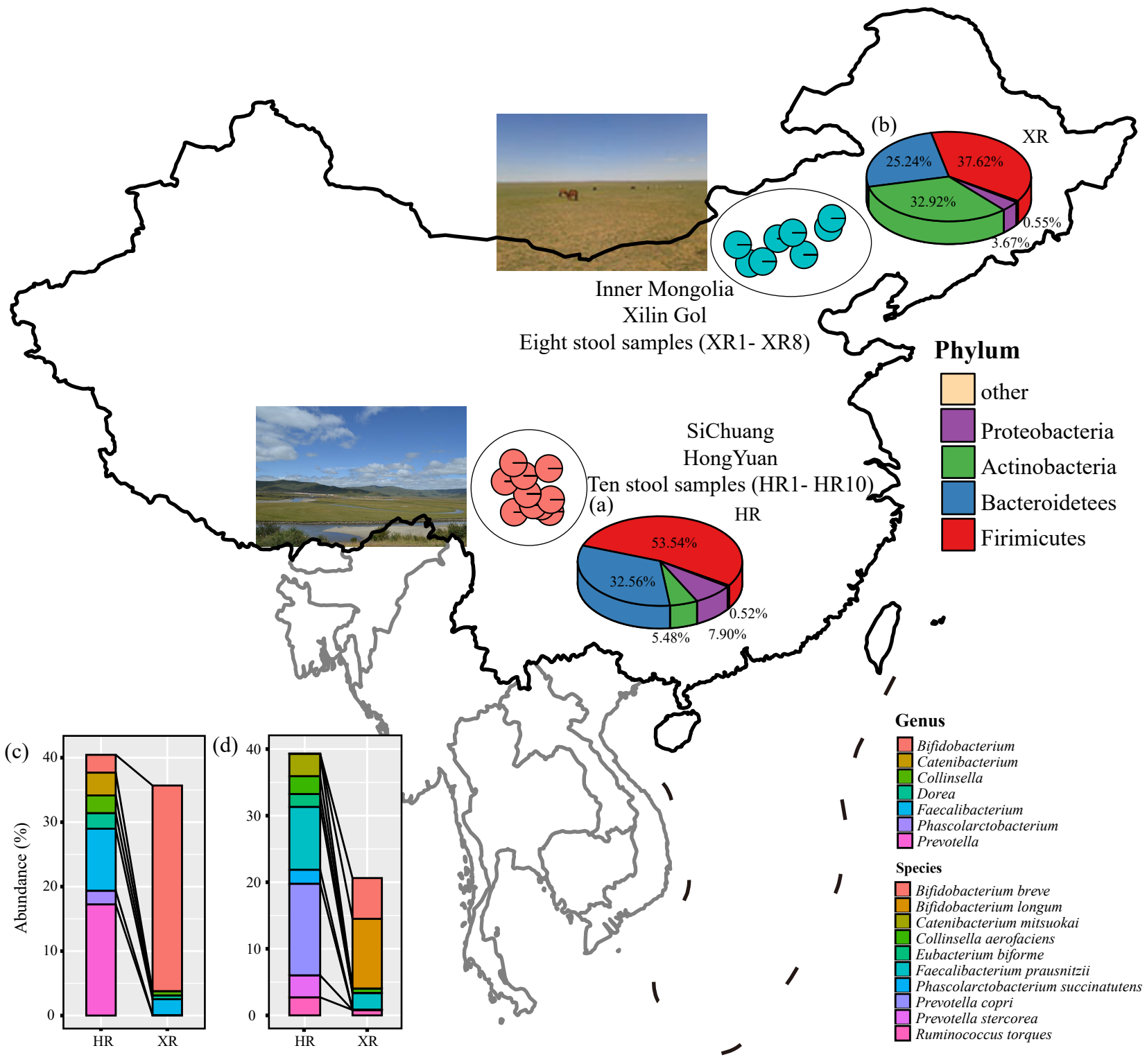

Supplement: Supplementary file 3 — Figure S1 [file FSN3-9-3258-s001.pdf]

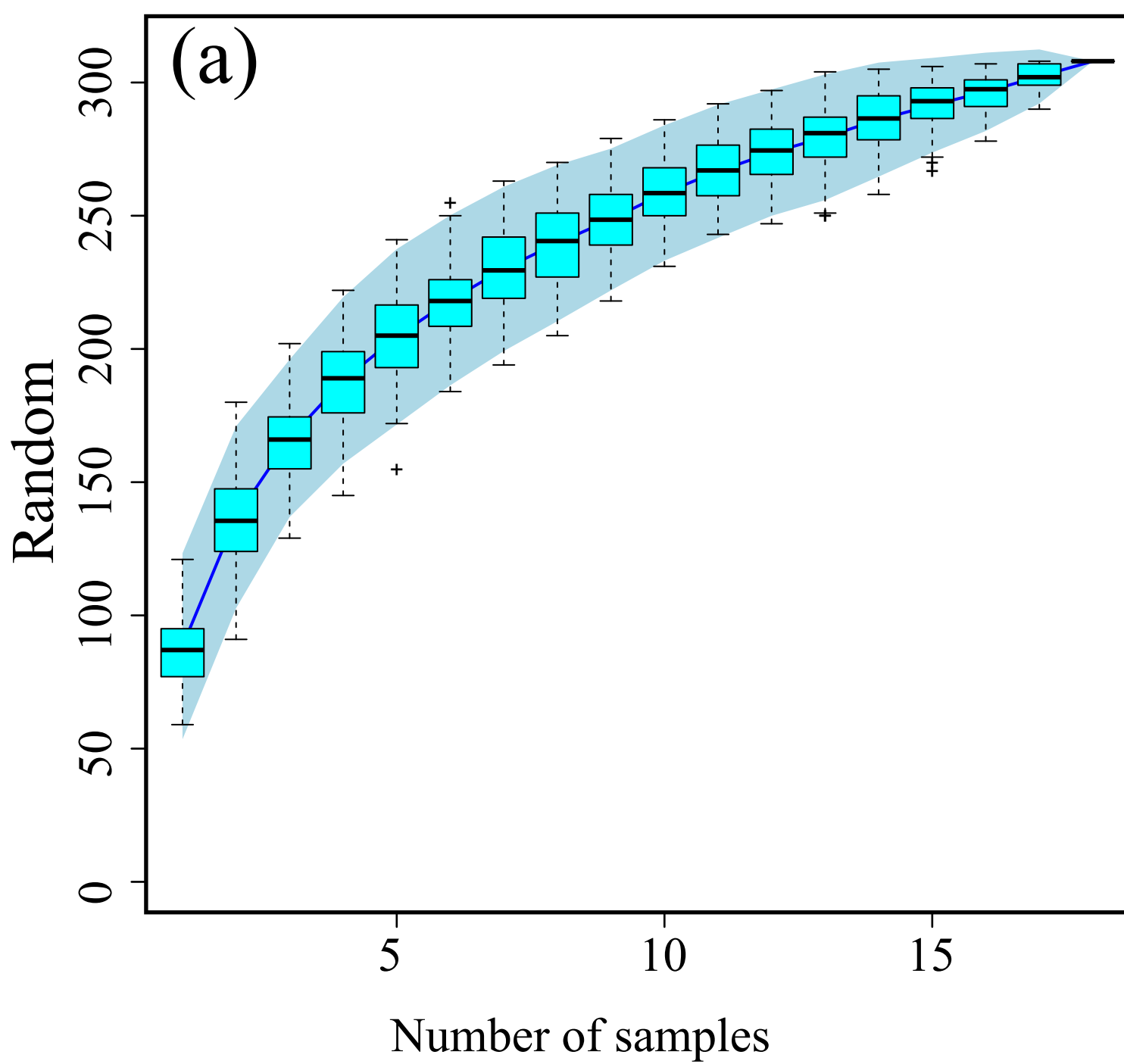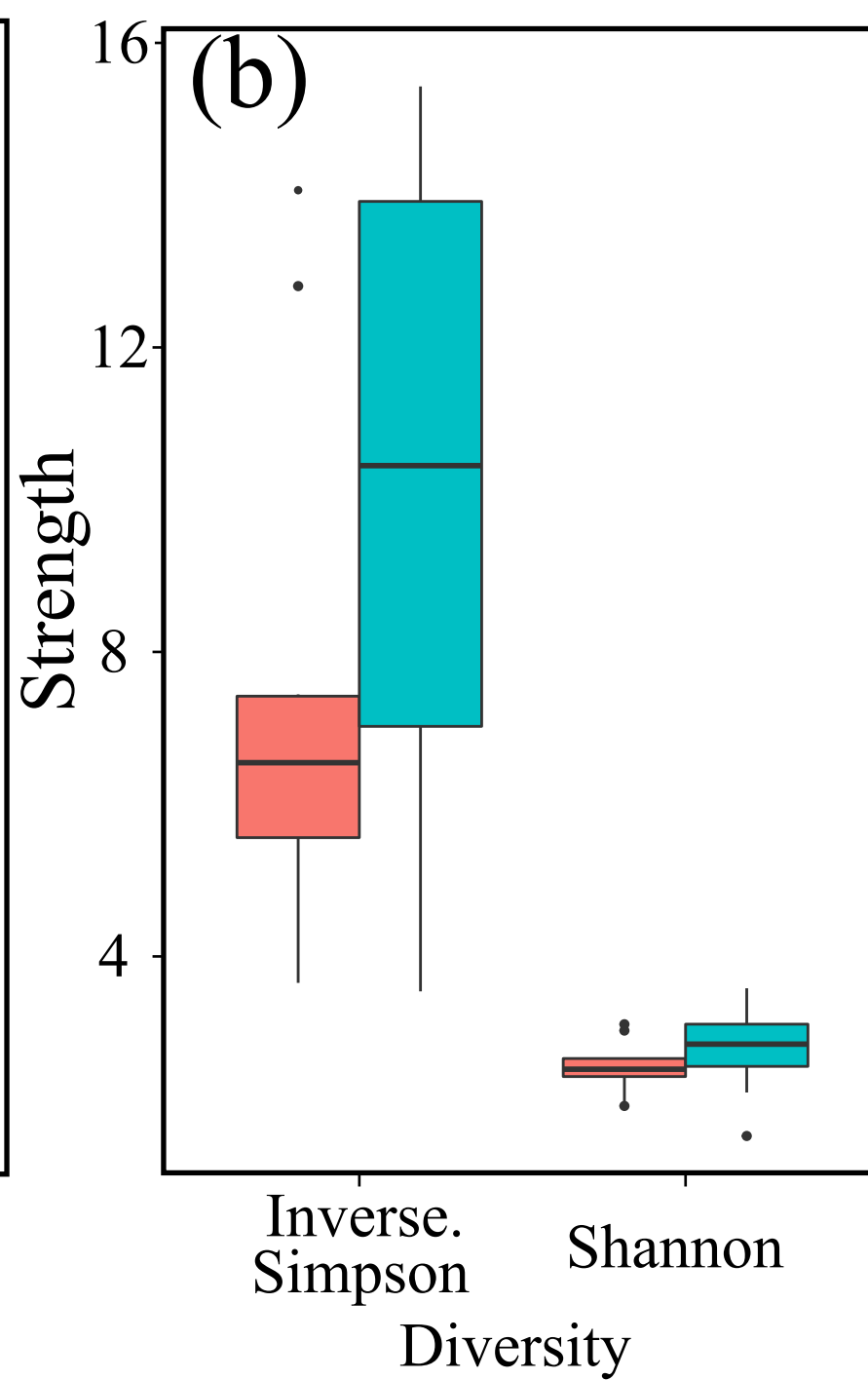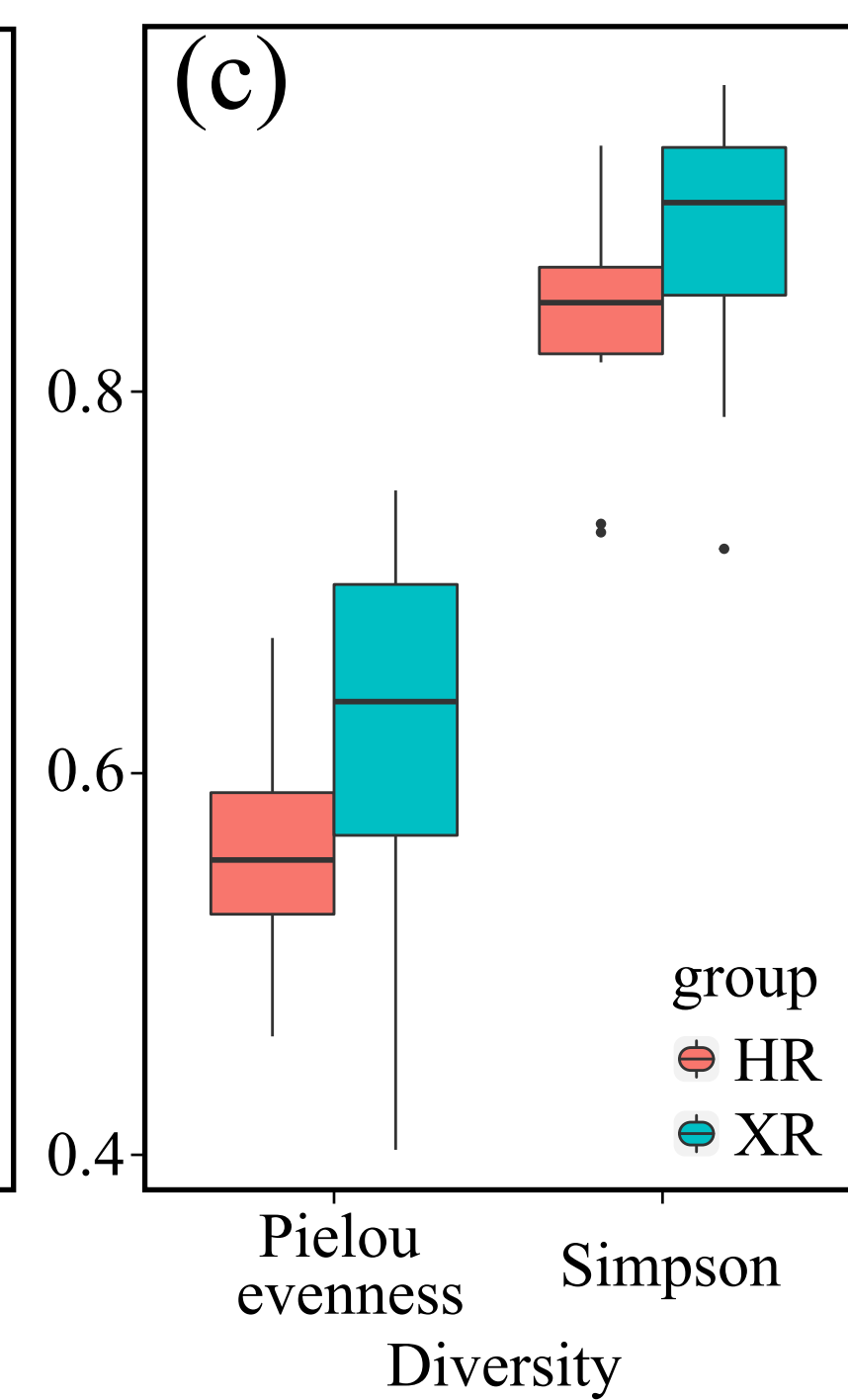

Supplement: Supplementary file 4 — Figure S2 [file FSN3-9-3258-s003.pdf]

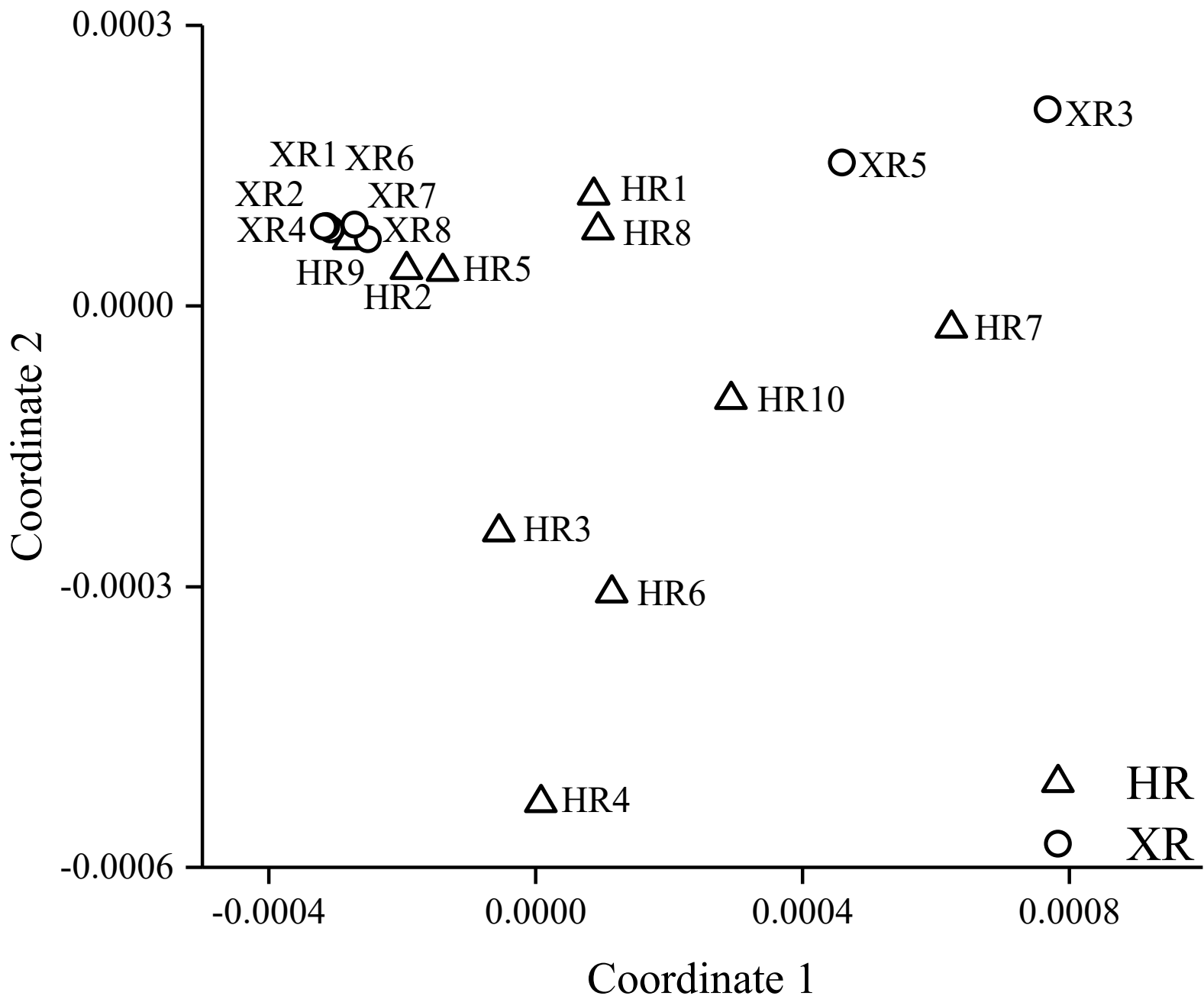

Supplement: Supplementary file 5 — Figure S3 [file FSN3-9-3258-s004.pdf]
